# CoAl-LDH decorated with cerium oxide as an efficient adsorbent for restoring low-concentration phosphate in wastewater

**DOI:** 10.1039/d3ra08201f

**Published:** 2024-03-27

**Authors:** Fengqin Tang, Hui Bai, Yahui Chen, Chunhui Shi, Dong Wang, Yaju Zhang, Wenyuan Liu, Ling Yang, Libing Hu

**Affiliations:** a Engineering Laboratory of Chemical Resources Utilization in South Xinjiang of Xinjiang Production and Construction Corps, College of Chemistry and Chemical Engineering, Tarim University Alar 843300 Xinjiang P. R. China 17797938871@163.com hlbin148@163.com; b Analysis and Testing Center, Tarim University Alar 843300 Xinjiang P. R. China

## Abstract

The requirement for the removal of phosphorus (P) from wastewater has become progressively stringent, therefore, it is essential to remove low-concentration phosphate from secondary effluents through a tertiary treatment. One of the biggest challenges in removing phosphate from wastewater is the development of low-cost, green, and pollution-free adsorbents. In this study, novel, eco-friendly and low-cost CeO_2_ nanosphere modifying CoAl-LDH nanosheets (CoAl-LDH/CeO_2_) were successfully fabricated using a classical hydrothermal strategy. The microstructure and morphology of CoAl LDH/CeO_2_ were characterized using SEM, TEM, FTIR, XRD, TG, XPS, and BET techniques. The performance of the P adsorption from water for CoAl-LDH/CeO_2_ was investigated. The influences of adsorption parameters, such as adsorbent dosage, pH, phosphate concentration, adsorption time, and experimental temperature, were investigated through batch adsorption experiments. The batch adsorption experiments showed that the P removal by CoAl-LDH/CeO_2_ could reach 93.4% at room temperature within 60 minutes. CoAl-LDH/CeO_2_ showed ultrafast and high-efficiency adsorption for low concentration P contaminated wastewater. Pseudo-second order model exhibited better fitting with the kinetics of the phosphate adsorption, while the Freundlich model well-described the isotherm results (*R*^2^ > 0.999). Although Cl^−^, NO_3_^−^and SO_4_^2−^ coexisted in the solution, CoAl-LDH/CeO_2_ still possessed favourable selectivity for phosphates. More importantly, the adsorption capacities of CoAl-LDH/CeO_2_ retained over 85% after five cycles. Therefore, the low cost and sustainable utilization of CoAl-LDH/CeO_2_ for the phosphate removal from secondary effluent with phosphate at a low concentration highlights its potential application to alleviate eutrophication.

## Abstract 2

The requirement for the removal of phosphorus (P) from wastewater has become progressively stringent, therefore, it is essential to remove low-concentration phosphate from secondary effluents through a tertiary treatment.

## Graphical abstract

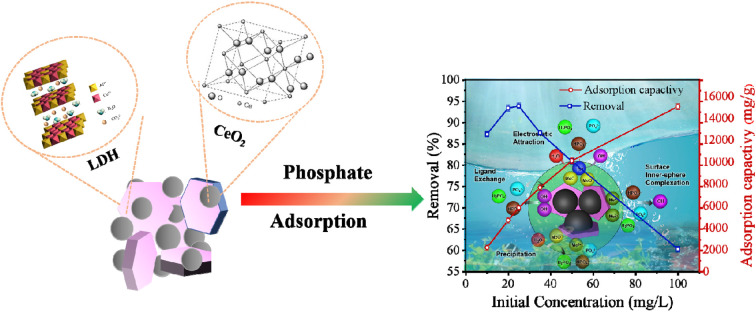

## 1. Introduction

In recent decades, the widespread pollution of the Earth's environment caused by human activities and the negative impact on humans and ecosystems have attracted widespread attention.^1^ Phosphorus, as one of the most abundant resources in nature, is an essential element for humans and other living organisms.^2^ Phosphate, as a key factor in the growth of aquatic organisms, plays an important role in the biological life cycle.^3^ However, as a result of human activities, the extreme usage and direct discharge of phosphate has led to an increased phosphate concentration in lakes, resulting in unimaginable eutrophication of lakes and rivers. Studies have shown that if phosphate concentration exceeds 0.03 mg L^−1^ in a waterbody, red tide or algae bloom may occur, which can seriously reduce water quality and damage the function of the water ecosystem, and even seriously endangered human health and survival of other organisms.^4^ Consequently, in order to control water pollution, increasingly strict requirements have been put forward for phosphorus emissions. Specifically, China currently allows a maximum phosphorus content of 0.5 mg P L^−1^ in the secondary effluent of sewage treatment plants, which has replaced the previous 1.0 mg P L^−1.^ Therefore, to obtain satisfactory low-level phosphate, the secondary effluent requires further phosphate treatment.^5^ Currently, there are many existing phosphorus removal treatment technologies, including chemical precipitation, biological methods, electrodialysis, membrane separation and adsorption.^6^ Among them, the adsorption method is one of the most desirable technologies for phosphate removal by virtue of its wide applicability, simple process and stabilizing effect.^7^ However, it is key to devise an adsorbent with excellent adsorption performance, favourable regeneration property and distinctive selectivity to efficiently remove phosphate. Until now, the large variety of adsorbents, encompassing inorganic adsorbents (bentonite, attapulgite mesoporous silica nanoparticles, activated carbon)^8–13^ and inorganic-organic hybrid materials (Ce-MOF, ZIF-8) *etc*,^14,15^ have been extensively investigated and applied in the purification of effluent. However, these research studies primarily revolved about relatively high initial phosphorus concentrations over 20 mg L^−1^, Therefore, the development of adsorbents with high-adsorption capacity for the initial phosphorus at lower concentrations is urgent and of great significance which is not representative of the wastewater quality. And the above-mentioned adsorbents have drawbacks, such as poor reusability, high cost and weak selectivity, *etc*, making it stiff to employ to the phosphate adsorption process. These issues conspicuously restrict the large-scale production and application of phosphate adsorbents.^16^ Therefore, exploiting novel adsorbents with low cost is urgent for removing low-concentration phosphate.

In recent years, rare earth elements, especially lanthanum (La) and cerium (Ce), have gradually gained attention in the selective phosphate adsorption because of their low cost, biocompatibility, thermal stability, and non-toxic or low toxicity to the human body.^17^ Among different kinds of rare earth elements, cerium (Ce) is the amplest in crust of the earth. As well known, China is not only rich in rare earth resources, but also has a complete range of minerals. In particular, rare earth mineral resources are rich in the Great Basin of Tarim in Southern Xinjiang. Under the national “the Belt and Road” initiative, the development of rare earth resources has important practical significance for promoting the economic development of Xinjiang. Until now, some novel CeO_2_-based sorbents have been exploited recently to remediate water containing phosphate. For instance, Liu *et al.* successfully prepared CeO_2_ decorated lignin (L-NH_2_) as a nanoadsorbents (L-NH_2_@Ce) through an unsophisticated approach and discovered that the phosphate adsorption capacity of L-NH_2_@Ce nanoparticles could reach 27.86 mg g^−1^.^18^ In addition, some relevant studies also have shown that CeO_2_ has a specific affinity for phosphates, due to its the strong alkalinity and low ion potential.^19,20^ Although CeO_2_ is considered as one of the most promising and attractive adsorbents for capturing phosphate, the pure CeO_2_ displays an unsatisfactory performance towards the phosphate removal. There remains much room in the development of Ce-based adsorbents for enhancing phosphate adsorption.

It has been reported that constructing CeO_2_-based composites with other materials, especially 2D materials, is an efficient protocol to promote the phosphate adsorption performance of CeO_2_. For example, CeO_2_-PRGO nanocomposite revealed remarkable adsorption performance.^21^ Hydroxides play an indispensable role in the geochemical cycle of metals and nutrients.^22^ Amongst all kinds of 2D materials, hydrotalcite and hydrotalcite-like compounds, as a kind of 2D anionic layered clay mineral, are universally known as layered double hydroxides (LDHs), which have brucite-like layers composed of divalent and trivalent metallic cations with interlayer anions and water molecules situating between the layers.^23^ LDHs with different elemental compositions and metal cation ratios can influence their appearance and crystallographic structure, enabling them to be designed for specific objectives.^24^ Therefore, LDHs have attracted widespread attention in numerous fields, such as catalysis,^25^ membrane separation,^26^ drug delivery,^27^ thanks to their large surface area, more animated adsorption sites, well-ordered layered structure, unique characteristics of environmentally friendliness, favorable anion exchange ability, and high controllability. Based on the as-mentioned advantages, LDHs have been considered as an ideal adsorbent candidate for alleviating phosphate pollution.^28,29^ Recently, many attempts have been committed to improve the phosphate removal capability of LDHs by forming complex materials in conjunction with other nanomaterials or incorporating a third metal into the LDH structure, which can provide higher specific surface area and more active adsorption sites.^30,31^ However, the composites of LDHs and CeO_2_ for the phosphate capture as an efficient adsorbent have been rarely reported.

In the present study, we developed CoAl-LDH/CeO_2_ nano-adsorbent by adopting a simple hydrothermal method for removing simulated secondary wastewater containing low concentration phosphate. The prepared adsorbent was further characterized by SEM, XRD, FTIR, XPS, TGA, BET, and Zeta potential to reflect feasibility of CoAl-LDH/CeO_2_ in phosphate adsorption by their morphology, crystal structure, surface properties, thermal stability and elemental distribution. Batch experiments were conducted to investigate the effects based on initial concentration, adsorption time, temperature, adsorbent dosage, solution pH and coexisting anions on phosphate adsorption efficiency. Additionally, the reusability of the CoAl-LDH/CeO_2_ nano-adsorbent was also investigated.

## 2. Materials and methods

### 2.1. Reagents

For the synthesis of CoAl-LDH/CeO_2_, the following analytical grade reagents were used:

Cobalt nitrate hexahydrate (Co(NO_3_)_2_·6H_2_O, McLean Chemical Co., Ltd), Aluminium nitrate (Al(NO_3_)_3_·9H_2_O, McLean Chemical Co., Ltd), Cerium nitrate hexahydrate (Ce(NO_3_)_2_·6H_2_O, Aladdin Chemistry Co., Ltd), Sodium hydroxide (NaOH, Tianjin Yongda Reagent Co., Ltd), Sodium carbonate (Na_2_CO_3_, Tianjin Yongda Reagent Factory), Ethylene glycol (C_2_H_6_O_6_, Aladdin Chemistry Co., Ltd), Ethanol (EtOH, Tianjin Fuyu Fine Chemical Co., Ltd) and potassium dihydrogen phosphate (KH_2_PO_4_) were obtained from Sinopharm Chemical Reagent, China. Ascorbic acid and ammonium molybdate were provided by Xi'an Sanpu Chemical Reagent Co., Ltd. Antimony potassium tartrate was supplied by Tianjin No. 4 Chemical Reagent Factory. Distilled water was self-made throughout the experiments. All reagents were unpurified.

### 2.2. Preparation of materials

#### 2.2.1. Preparation of materials

CoAl-LDH was prepared based on a facile hydrothermal method described previously.^32^ In a typical process, 3.7 g of Al (NO_3_)_3_·9H_2_O and 5.8 g of Co (NO_3_)_3_·6H_2_O (the molar ratio of Co to Al was 1 : 2) were added to 50 mL of deionized water, after which the resulting mixture was stirred to form a uniform solution that was considered as solution A. Meanwhile, solution B was obtained with the mixture of 0.01 mol Na_2_CO_3_ and 0.03 mol NaOH dissolving in 30 mL of deionized water after magnetic stirring, after which solution B was added drop by drop into solution A, and the entire system formed a uniform pink solution with the continuous magnetic stirring for 30 minutes. Subsequently, the as-obtained pink solution was transferred into an autoclave, which was kept at 120 °C for 6 h in an oven. After that, the pink product were centrifuged and washed three times by using the self-made deionized water. Finally, the desirable CoAl-LDH was collected after dried at 60 °C for 12 h before further use.

#### 2.2.2. Preparation of CoAl-LDH/CeO_2_ composite

In order to obtain the CoAl-LDH/CeO_2_ composite, 2.0 g of Ce (NO_3_)_2_·6H_2_O was firstly dissolved in 60 mL of ethylene glycol with ultrasonic treatment. After that, 4 mL of water was added. Then, 2.0 g of the as-prepared CoAl-LDH was dispersed into the mixed solution and strongly stirred for 40 min. Subsequently, the reaction compound was transferred to a 100 mL of Teflon-lined stainless-steel autoclave which was then heated to 180 °C for 3 h. After the temperature reached room temperature, the supernatant was discarded and the products were washed several times with ethanol and water. Finally, the remaining brown powders were dried in an oven with the heat temperature maintaining at 60 °C to acquire the CoAl-LDH/CeO_2_ sorbent. For comparison, the pure CeO_2_ was prepared without CoAl-LDH under the same condition.

### 2.3. Materials characterization

The produced materials (CoAl-LDH/CeO_2_, CoAl-LDH and CeO_2_) were characterized by the following analytical techniques: Scanning electron microscopy (SEM, Hitachi SU-70 feld-emission scanning electron microscope) with an energy of 5.0 KV and manipulating transmission electron microscopy (TEM) (a Germany-made ZEISS-MERLIN (GEMINI-2) instrument) for morphology analysis of the conflated samples, nitrogen adsorption/desorption isotherm by the Brunauer–Emmet–Teller (BET) and Barret–Joyner–Halenda (BJH) approaches to obtain the surface area and pore diameter of the adsorbents, Fourier Transform-Infrared spectroscopy (FT-IR) with the wavelength rang from 400 to 4000 cm^−1^, X-ray diffraction (XRD) to analyze the crystal structure of the materials, thermogravimetric analysis (TG) to investigate the degradation temperature of the materials. The compounds of the synthetic adsorbents were measured by X-ray photoelectron spectroscopy (XPS) spectra (Thermo Fisher Scientifc ESCALAB 250 spectrometer).

### 2.4. Adsorption tests

The adsorption capability of the as-obtained adsorbents (CoAl-LDH, CeO_2_ and CoAl-LDH/CeO_2_) was tested for the phosphate adsorption in aqueous solution using a range of batch adsorption experiments. 0.1 mol L^−1^ of HCl or NaOH solution was used to adjust the pH level of the solution. The effects of adsorbent dosage, contact time, initial phosphate concentration, solution pH, and coexisting cation concentration on the phosphate adsorption efficiency of CoAl-LDH/CeO_2_ were investigated through batch experiments. Adsorption isotherms of phosphate was conducted containing 0.1 g adsorbent and 25 mL different concentration phosphate (20–100 μg mL^−1^) in a 100 mL flask, the entire system was reacted for 60 min at 25 °C. The adsorption kinetics experiment was carried out in the mixture of 0.1 g of the adsorbent with 25 mL of 20 μg mL^−1^ phosphate solutions. The solution after adsorption was collected at different time intervals. The mixture solution was filtered by employing a nylon membrane (0.45 μm) from the solution, and the filtrate was determined through manipulating molybdenum blue-ascorbic acid spectrophotometric method (*λ* = 700).

The adsorption capacity and its corresponding removal efficiency were respectively determined according to the following eqn (1) and (2):1
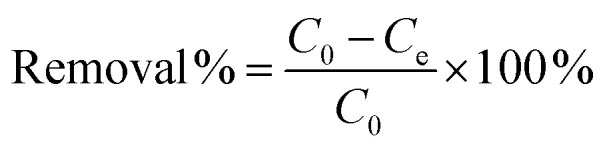
2
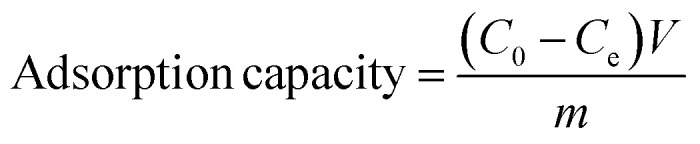
In which *C*_0_ was initial phosphate concentrations (mg L^−1^), Ce represented equilibrium phosphate concentrations (mg L^−1^), *q*_e_ denoted adsorption capacity (mg g^−1^) at equilibrium condition, *m* stood for the mass of adsorption samples (g), and *V* was volume of phosphate solution (L), respectively.

### 2.5. Influence test of coexisting ions and regeneration experiments

There are a large number of anions in water, such as carbonate, bicarbonate, nitrate, sulfate, chloride, *etc.*, which may prevent the phosphate removal during the adsorption process. Therefore, the influence of these anions (SO_4_^2−^, NO_3_^−^, HCO_3_^−^ and CO_3_^2−^) was investigated. To examine the influence of coexisting anions on phosphate adsorption, 0.1 g of CoAl-LDH/CeO_2_ was added into 20 μg mL^−1^ phosphate solutions with 0.01 M of each different anion like SO_4_^2−^, HCO_3_^−^, NO_3_^−^, and CO_3_^2−^, respectively. The compounds were shaken at 25 °C for 60 min. The regeneration and reusability of CoAl-LDH/CeO_2_ adsorbent were studied. In short, CoAl-LDH/CeO_2_ was introduced into KH_2_PO_4_ solution (20 μg mL^−1^), followed by shaking at 25 °C for 2 h. After that, the adsorbed materials were assembled by applying a 0.5 M NaOH solution and then dried after the constant oscillation (6 h) for the next recycling experiment. The removal rates of adsorbents were compared and analyzed after repeated experiments.

## 3. Results and discussion

### 3.1. Fabrication and characterization of CoAl-LDH/CeO_2_

**Fig. 1:**
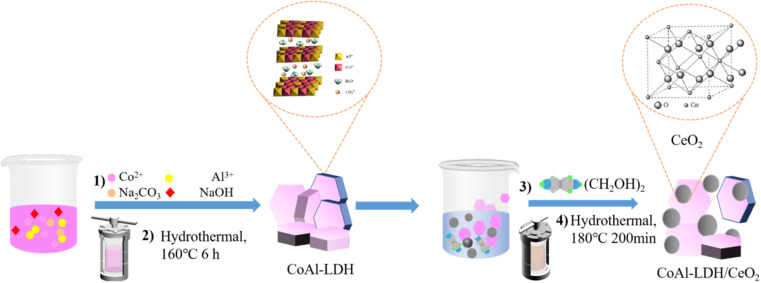
Schematic process for the facile preparation of CoAl-LDH/CeO_2_.

Fig. 1 displayed the simple preparation process of nano adsorbent materials (CoAl-LDH/CeO_2_). Firstly, nanosheet CoAl-LDH was obtained through hydrothermal reaction, and the surface of LDH was positively charged. Then CoAl-LDH was dispersed in Cerium dioxide precursor solution, CeO_2_ nanoparticles are synthesized *in situ* on hydrotalcite through the hydrolysis of Ce^3+^. CoAl-LDH and CeO_2_ were bonded together through electrostatic interactions. By centrifugal washing, LDH modified by CeO_2_ nanoparticles (CoAl-LDH/CeO_2_) were obtained.

### 3.2. Morphology and structure analysis

#### 3.2.1. SEM and TEM micrograph examination

**Fig. 2:**
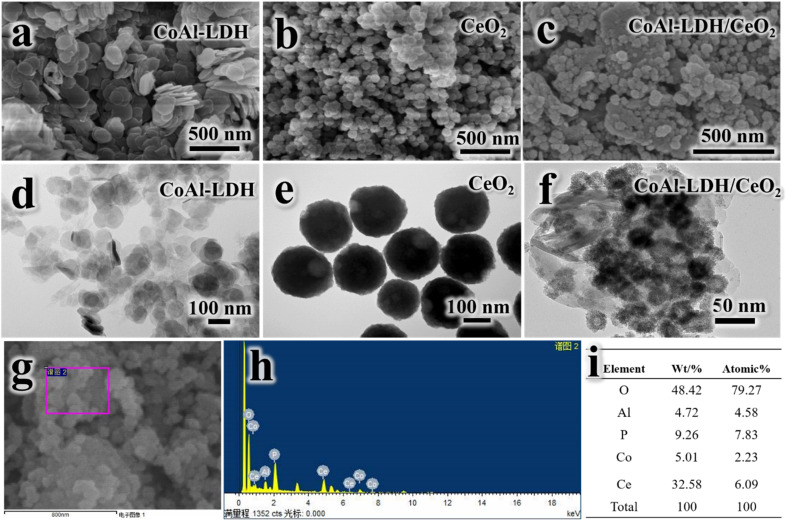
SEM micrograph for CoAl-LDH (a), CeO_2_ (b) and CoAl-LDH/CeO_2_ (c), TEM micrograph for CoAl-LDH (d), CeO_2_ (e) and CoAl-LDH/CeO_2_ (f), element composition and SEM-EDX analysis spectrums of CoAl-LDH/CeO_2_ (g–i).

The morphology and microstructure of CoAl-LDH, CeO_2_ and CoAl-LDH/CeO_2_ was characterized by employing the transmission electron microscope (TEM). As exhibited in Fig. 2a, the piled hexagonal and separated CoAl-LDH nanosheets (Fig. 2d) with a diameter of 70 to 150 nm could be clearly investigated. The thickness of the sample was about 10–20 nm, meanwhile it was relatively flat with relatively uniform particle size with poor contrast and slight agglomeration between extremely fine particles, which were regular and well-defined and exhibited typical lamellar structure similar to those reported in previous publications.^33^ It could be seen in Fig. 2b that the obtained CeO_2_ sample displayed smaller particles with a diameter of 150 to 200 nm and were relatively spherical with uniform size distribution (Fig. 2e). Furthermore, the distribution in size was homogeneous with relatively little aggregation between particles and the shape appeared round. As shown in Fig. 2c, the addition of CoAl-LDH into the CeO_2_ combination could induce the formation of nanospherical shape with smaller diameter (30–80 nm) and CeO_2_ for increasing the adsorption surface area and active sites. It was obvious that cerium(iv) oxide nanoparticles were hollow spheres (Fig. 2f). Meanwhile, it could be clearly seen that the segmental surface of CoAl-LDH nanosheets were attached with circular CeO_2_, indicating that CoAl-LDH and CeO_2_ were successfully combined to form CoAl-LDH/CeO_2_. With the addition of CoAl-LDH, the higher agglomeration degree increased in CoAl-LDH, because the OH- ligand derived from (CH_2_OH)_2_ made the metal hydroxide precursor agglomerate under hydrothermal conditions. Ce^4+^ or Ce^3+^ hydroxides experienced an attack by basic medium to dissolve and reacted at high temperature and pressure during the hydrothermal treatment,^34^ after which it precipitated in the form of insoluble CeO_2_ particles.^35^ We speculated that the dissociation of Ce (OH)_2_ and the presence of hydrotalcite might prevent the CeO_2_ grains from growth and decrease the particle size to a smaller nanometer range. Additionally, In Fig. 2h, four elements, such as O, Al, Co and Ce, were evenly distributed in the composite, which indicated that the compounding of CeO_2_ and CoAl-LDH was successful. To sum up, as can be seen in Fig. 2(g–i), It was evident that the CoAl-LDH/CeO_2_ composite remained composed of O, Al, Co and Ce. Furthermore, EDX map scanning (Fig. 2h and i) of phosphate-attached CoAl-LDH/CeO_2_ indicates that the P element is evenly dispersed over the sample.

#### 3.2.2. XRD analysis

**Fig. 3:**
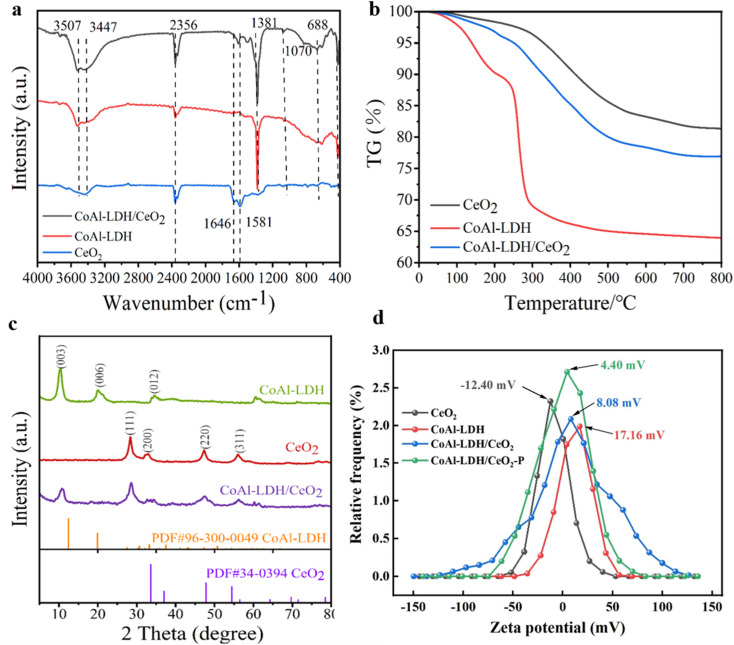
(a) FT-IR spectra of CoAl-LDH, CeO_2_ and CoAl-LDH/CeO_2_. (b) TG curves of CoAl-LDH, CeO_2_ and CoAl-LDH/CeO_2_. (c) XRD patterns of CoAl-LDH, CeO_2_ and CoAl-LDH/CeO_2_. (d) Zeta potential of the CoAl-LDH, CeO_2_, CoAl-LDH/CeO_2_ before and after phosphate adsorption curves.

The phase structure of the as-prepared samples of CoAl-LDH, CeO_2_ and CoAl-LDH/CeO_2_ was investigated by XRD, as depicted in Fig. 3c. The pattern of CoAl-LDH represented sharp and clear reflections, indicating that the CoAl-LDH phase formed a well crystallized layered structure in the form of carbonates. The crystal planes of CeO_2_ with a globular structure were, respectively, observed at 28.4°, 32.8°, 47.3° and 56.1° that were corresponding to the (111), (200), (220) and (311) diffraction planes of CeO_2_ (PDF#34-0394).^36^ Five obvious peaks located at 10.4°, 20.1°, 34.6°, 60.6° and 61.5° could be detected indexing to the (003), (006) and (012), (110) and (113) planes crystal planes,^37^ which could be corresponded to CoAl-LDH (PDF#96-300-0049). Meanwhile, it could be seen that the XRD line of the adsorbent clearly characterized the diffraction peak with a distinct CeO_2_ characteristic peaks. The crystal planes of CoAl-LDH/CeO_2_ were observed at 2θ values of 28.6°, 32.7°, 47.5° and 56.2°, respectively, corresponding to the (111), (200), (220) and (311) diffraction planes of the pure CeO_2_. The 2*θ* values of 10.7°, 21.0°, 34.3°, 60.6° and 61.5°, respectively, were indexed to the (003), (006) and (012), (110) and (113) diffraction planes of CoAl-LDH. The crystal structure of CeO_2_ had kept well after introducing CoAl-LDH. Whereas, in CoAl-LDH/CeO_2_, it was found that the characteristic peak of CoAl-LDH became weaker compared to pure material, which might be related to the encapsulation of some CoAl-LDH into a large amount of spherical CeO_2_. In addition, no impurity peaks except for CeO_2_ and CoAl-LDH were watched in CoAl-LDH/CeO_2_ according to the XRD spectrum, indicating that no by-products were produced.

#### 3.2.3. FTIR analysis

The FT-IR spectra for the samples of CoAl-LDH, CeO_2_ and CoAl-LDH/CeO_2_ were demonstrated in Fig. 3a. As well known, they basically had the distinctive functional groups on the surface. In case of pristine CoAl-LDH, the broad and intense peak absorption peak at about 3600∼3100 cm^−1^ could be indexed to the functional groups of OH- on the surface of CoAl-LDH and the vibrations of O–H bonding in the adsorbed interlayer water molecules,^38^ while the absorption peak at 1381 cm^−1^ was thanks to the stretching vibration of CO_3_^2−^ anions. The absorption peaks at 618 cm^−1^ and 426 cm^−1^ could be resulted in the metal O bond (M–O) composed of Al–O and Co–O ion bonds.^39^ In case of p CeO_2_, the broad peaks at 3429 and 1641 cm^−1^ were attributed to the stretching vibration of –OH groups, because of the absorbed H_2_O molecules. The peaks around 1641 cm^−1^ might stem from y(CeO/C=O) or y(OH–) which could be adsorbed during the resultant of ball-type CeO_2_ from metal–glycol ions to pure ceria, obviously indicating the surface modification of CeO_2_ during the synthetic procedure.^40^ The peaks at 1616 cm^−1^ and 1380 cm^−1^ was attributed to the asymmetric stretching vibrations mode of C=O related to carbonate and carboxylate. Furthermore, the peak at approximately 1070 cm^−1^ could be indexed to the C–OH stretching vibration. These characteristic peaks validated the presence of ethylene glycol on the CeO_2_ particles. Peaks located below 1000 cm^−1^ were attributed to the stretching vibration of Ce–O–Ce bond.^41^ All of the characteristic peaks of CeO_2_ and CoAl-LDH could be seen in the resulting CoAl-LDH/CeO_2_ composite. The FT-IR spectra further indicated that CeO_2_ spheres were formed on the surface of CoAl-LDH nanosheets, meanwhile strong electrostatic bonding interaction between them was generated, which might be great significant for improving the performance of phosphate adsorption.

#### 3.2.4. TG analysis

To acquire further information of the ingredient and phase transformation of the synthetic samples. The thermal degradation behaviors of CoAl-LDH, CeO_2_ and CoAl-LDH/CeO_2_ were investigated by thermogravimetric analysis. The TGA curves of CoAl-LDH, CeO_2_ and CoAl-LDH/CeO_2_ were depicted in Fig. 3b. Clearly, the TGA curve of the CoAl-LDH powder exhibited three stages of mass loss. The first stage of 9.8% weight loss from 45 to 209 °C was the desorption of the physic- and chemi-sorbed water. The 21% of mass loss took place with the temperature at 130 °C ∼300 °C could be assigned to the subsequent liberation of carbonate ions and dehydroxylation of the CoAl-LDH structure. In addition, the 5% of mass loss above 300 °C was owing to further dehydroxylation from CoAl-LDH layers.^42^ For CeO_2_ particles, the pyrolysis process was divided into a two-step pattern. The first stage of weight loss was due to the desorption of physically adsorbed and ethylene glycol molecules with the temperature ranging from 25 to 300 °C, amounting to about 3.7%. The second stage confirmed the removal of ethylene glycol molecules from 300 °C to 800 °C, and the total weight loss was about 12.2%. While the temperature attained a certain level, the organic groups and CeO_2_ nanocrystals would break down, which just illustrated the final phase above 300 °C. As for the curve of CoAl-LDH/CeO_2_ hybrids, it integrated the features of the other two curves. The mass loss of CeO_2_ from room temperature to 800 °C was decreased, which confirmed CoAl-LDH successfully combined with CeO_2_ as modifiers and intensified its thermal stability.

#### 3.2.5. Zeta potential analysis

Zeta potential could be employed to characterize the surface charge state of materials. As exhibited in Fig. 3d, the Zeta analysis diagrams of CeO_2_ (a), CoAl-LDH (b) and CoAl-LDH/CeO_2_ (c) with phosphate adsorption were exhibited. From the Fig. 3d, it could be seen that the surface of CoAl-LDH was positively charged with the highest value and a potential of about ∼17.16 mV, while CeO_2_ presented a mean zeta potential about ∼12.4 mV. Obviously, their zeta potential was opposite, which was of great benefit to the formation of heterojunctions both CoAl-LDH and CeO_2_ due to the Coulomb electrostatic interaction between them.^43^ For CoAl-LDH/CeO_2_, its surface was positively charged with a potential value of approximately 8.08 mV, which indicated that the CoAl-LDH and CeO_2_ were closely integrated by electrostatic force and the potential was decreased compared to hydrotalcite, conducive to adsorption between them.^44^ In addition, due to the enhanced adsorption of phosphorus by electrostatic attraction, the potential value changed to 4.4 mV after the adsorption of phosphate ions, indicating that CoAl-LDH/CeO_2_ had successfully adsorbed phosphate. All the above results indicated the successful preparation of CoAl-LDH/CeO_2_, which had the ability to remove phosphate.

#### 3.2.6. XPS analysis

**Fig. 4:**
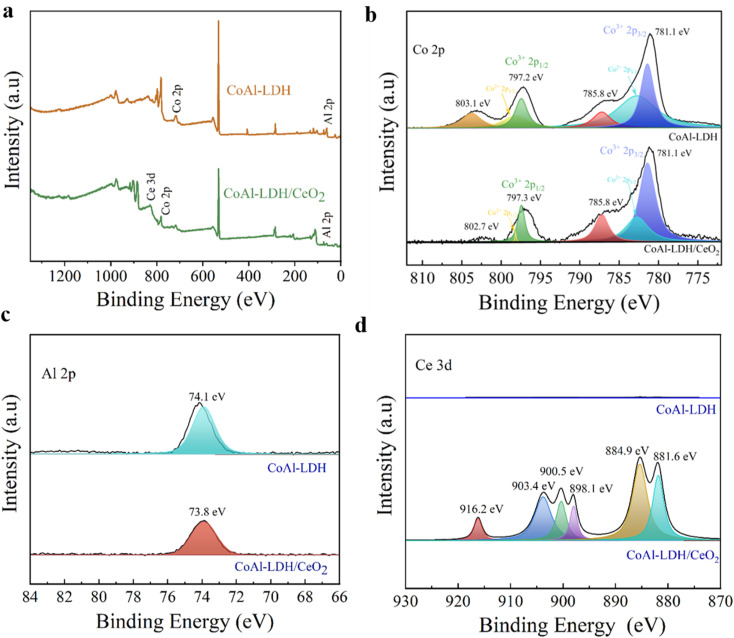
(a) XPS full spectrum of CoAl-LDH and CoAl-LDH/CeO_2_. High resolution spectra of (b) Co 2p, (c) Al 2p, and (d) Ce 3d of CoAl-LDH and CoAl-LDH/CeO_2_, respectively.

XPS spectroscopy was confirmed to research the chemical states of Co, Al, and Ce elements in CoAl-LDH/CeO_2_ and the possible chemical interactions between CeO_2_ nanospheres and CoAl-LDH nanosheets. As shown in Fig. 4, the measured XPS spectra of pure CoAl-LDH and CoAl-LDH/CeO_2_ sample were demonstrated. As it can be clearly seen, CoAl-LDH/CeO_2_ displayed the peaks corresponding to Co, Al, O, Ce elements well agreed with the XRD result. For bare CoAl-LDH, the high-definition Co 2p spectrum respectively substantiated the characteristic 2p3/2 and 2p1/2 constituents at 782.8 and 798.2 eV, because of the oxidation of Co. Besides, there were two clear small peaks appearing at 803.1 and 785.8 eV (Fig. 4b), which exhibited high-spin divalent Co^2+^ atoms. Moreover, the other peaks at 781.1 and 797.2 eV were related to the Co 2p3/2 and Co 2p1/2 of Co^3+^, indicating that Co^2+^ and Co^3+^ co-exist in the CoAl-LDH sample.^45^ While the high-resolution Al 2p spectrum had a binding energy of 74.1 eV on the 2p orbital, confirming the presence of Al^3+^ ions in CoAl-LDH. Furthermore, for CoAl-LDH/CeO_2_, it could be observed (see the Fig. 4b) that Co 2p spectrum displayed two peaks at 782.8 eV and 798.2 eV, respectively, which were indexed to orbitals of Co 2p3/2 and Co 2p1/2. In addition, two small peaks showed an appearance at 785.8 eV and 802.7 eV. They were the Al binding energy in the 2p orbital (Fig. 4c). the other peaks at 781.1 and 797.3 eV were related to the Co 2p3/2 and Co 2p1/2 of Co^3+^, indicating that Co^2+^ and Co^3+^ co-exist in the CoAl-LDH/CeO_2_ sample.

As demonstrated in Fig. 4d, the Ce 3d spectrum in CoAl-LDH/CeO_2_ could be divided into six peaks, and three peaks located at 881.6, 885.5, 884.9 eV attributing to Ce 3d5/2, whereas another three peaks Ce 3d3/2 were presented at 916.2, 903.4, and 900.5 eV, confirming that Ce^4+^ was the primary valence of Ce species in CeO_2_. Because oxygen vacancies presented in CoAl-LDH/CeO_2_, which served chances for the co-existence of Ce^3+^ and Ce^4+^.^46^ In addition, it was interesting that the binding energy of single substances and complexes had undergone varying degrees of change, thus the bonds between a single substance and complex were both physical bonds and chemical bonds. This once again revealed the stability of the adsorbents.^47^

#### 3.2.7. N_2_ adsorption–desorption analysis

**Fig. 5:**
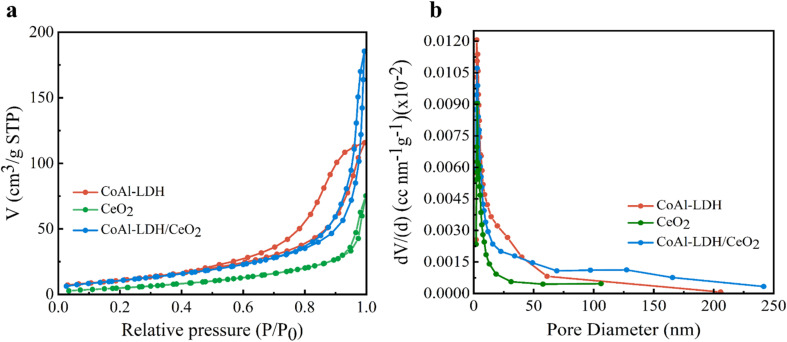
(a and b) Nitrogen adsorption/desorption isotherms of CoAl-LDH, CeO_2_ and CoAl-LDH/CeO_2_.

In order to get an insight into a deeper understanding of the structural properties of adsorbents, N_2_ adsorption/desorption isotherms and pore size distribution measurements for CoAl-LDH, CeO_2_ and CoAl-LDH/CeO_2_ were conducted. From inspection in Fig. 5a, it could be clearly observed that the N_2_ adsorption/desorption isotherms of the three samples manifested typical IV isotherms with H_3_ hysteresis loops,^48^ which indicated an evident characteristic of microporous materials. The BET surface areas of CoAl-LDH and CeO_2_ were 42.1291 and 21.3492 m^2^. g^−1^, respectively (Table 1), whereas that of CoAl-LDH/CeO_2_ was 41.4240 m^2^ g^−1^, which was less than that of CoAl-LDH. The increased specific surface area of CoAl-LDH/CeO_2_ could be primarily due to the growth of CeO_2_ with smaller particle size. The CoAl-LDH/CeO_2_ offered the biggest pore volume among the three samples (Table 1). Moreover, the pore size distribution of the CoAl-LDH/CeO_2_ (∼24.75 nm) was larger than that of CoAl-LDH (∼18.88 nm) and CeO_2_ (∼16.64 nm), as shown in Fig. 5b. Therefore, the N_2_ adsorption/desorption results showed that the composite might provide more adequate binding sites during adsorption, thus enhancing the phosphate adsorption performance. The pore diameter distribution further indicated that the CoAl-LDH/CeO_2_ displayed a mesoporous structure. In addition, the nanosheet structure of the 2D layered hydrotalcite had benefit to establishing maximum contact with the CeO_2_, which increased the adsorption effect of the combination.

**Table 1:** Pore structure parameters of CoAl-LDH, CeO_2_, CoAl-LDH/CeO_2_

	CoAl-LDH	CeO_2_	CoAl-LDH/CeO_2_
Specific surface area (m^2^ g^−1^)	42.12	21.34	41.42
Pore volume at *P*/*P*_0_ (cm^3^ g^−1^)	0.1753	0.1257	0.3014
Pore diameter (nm)	18.88	16.64	24.75

### 3.3. Adsorption isotherms, kinetics

At first, the phosphate adsorption isotherms on CoAl-LDH/CeO_2_ were investigated at room temperature. At a constant temperature, the adsorption isotherm defines the relationship between the quantity of phosphate adsorbed in the solution at equilibrium and its equilibrium concentration.

Canonical adsorption models were generally applied to depict the equipoise between the adsorbent contaminants and their concentration in solution at an invariant temperature. Nonlinear fitting was performed on the relationship between residual concentration and adsorption capability using the Freundlich and Langmuir isothermal models. An isotherm was drawn between the phosphate absorption (*q*_e_) of the CoAl-LDH/CeO_2_ and the equilibrium phosphate concentration (*c*_e_) in the solution. The adsorption isotherm provided detailed data on the adsorption efficiency of the adsorbent. Langmuir and Freundlich isotherms were most commonly used to evaluate the adsorption mechanism and interaction between adsorbents and adsorbate. The Langmuir isotherm adsorption model depicted the monolayer retention of adsorbents on a uniform surface. The Freundlich isotherm model was employed to depict multi-facet adsorption on sorbents with a heterogeneous surface.^49^

Isothermal adsorption equation of CoAl-LDH/CeO_2_ for phosphate was obtained according to eqn (3) and (4):3
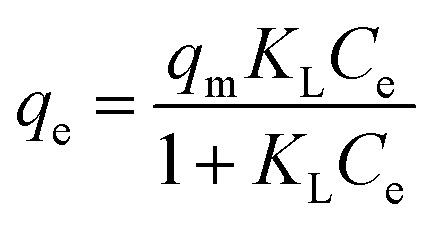

4 *q*_e_ = *K*_f_*C*_e_^1/*n*^

where *q*_e_ and *q*_m_ are the amount of solute absorbed by adsorbent per unit weight and the maximum adsorption capability (mg g^−1^), respectively. *C*_e_ is the concentration at equilibrium (mg L^−1^), *K*_L_ is the Langmuir isotherm constant which rigorously varies as a function of surface coverage on account of changes in adsorption heat (L mg^−1^), *K*_f_ (L g^−1^) stands for a measure of uptake capacity, and *n* indicates the interactivity between uptake sites on the adsorbent and contaminants.

**Fig. 6:**
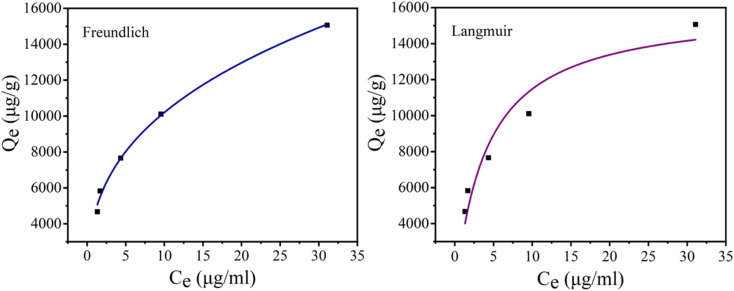
Adsorption isothermal model of phosphate by CoAl-LDH/CeO_2_.

And the results were displayed in Fig. 6 and Table 2. The probability of collision between CoAl-LDH/CeO_2_ and phosphate raised as the equilibrium concentration of phosphate increased, making full use of the adsorption sites. Therefore, the adsorption capacity of CoAl-LDH/CeO_2_ for phosphate also increased with the decrease of phosphate concentration. While the equilibrium concentration of phosphate increased, the probability of collision between CoAl-LDH/CeO_2_ and phosphate increased, and the adsorption sites were fully utilized. The *R*^2^ value of the Freundlich model (0.9947) was greater than that of the Langmuir model (0.9162), which exhibited that the adsorption process of CoAl-LDH/CeO_2_ for phosphate accorded with Freundlich model. The Freundlich isotherm parameter (1/*n*) ranged from 0.1 to 0.5, indicating that the adsorption reaction was relatively easy. All of these indicated that multi-layer adsorption on heterogeneous surfaces dominated the P uptake process of the CoAl-LDH/CeO_2._

**Table 2:** Isotherms parameters of phosphate onto CoAl-LDH/CeO_2_

Isotherm	Langmuir			Freundlich		
Parameters	*q*_m_ (mg g^−1^)	*K*_L_ (L mg^−1^)	*R* ^2^	*K*_f_ (mg g^−1^ (L mg^−1^)^1/*n*^)	1/*n*	*R* ^2^
	16.03	0.252	0.9162	4.597	0.3461	0.9947

Secondly, the adsorption kinetics of CoAl-LDH/CeO_2_ on phosphate were studied to assess the adsorption efficiency of the adsorbent and understand the adsorption mechanism. To appraise adsorption rate of CoAl-LDH/CeO_2_, the impact of adsorption time on adsorption kinetics of phosphate adsorption was investigated at a certain phosphate concentration. Furthermore, the kinetic data was fitted by pseudo-first- order and pseudo-second-order models. Here, the pseudo-first-order model elucidated that the amount of occupied sites was proportionate to the number of unoccupied sites, yet the pseudo-second-order kinetic model presumes a chemical reaction mechanism in which the adsorption rate was controlled through chemical adsorption by electron sharing or interchange between the adsorbate and the adsorbent. The adsorption capacity of phosphate (*q*_*t*_) was calculated using different models. The adsorption capacity of phosphate (*q*_*t*_) was calculated using different models,^50,51^ pseudo-first-order (eqn (5)) and pseudo-second-order models (eqn (6)), respectively.

5 *q*_*t*_ = *q*_e_(1−exp^−*k*_1_*t*^)

6
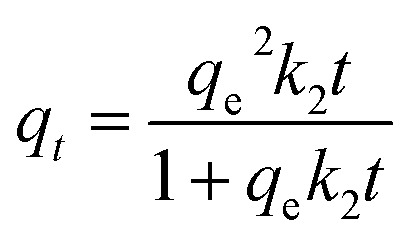
In which *q*_e_ represents the amounts of phosphate adsorption (mg g^−1^) at equilibrium, *q*_*t*_ is the amounts of the adsorbed phosphate (mg g^−1^) at different times (min), *k*_1_ is the pseudo-first-order rate parameter and *k*_2_ denotes the pseudo-second-order.

**Fig. 7:**
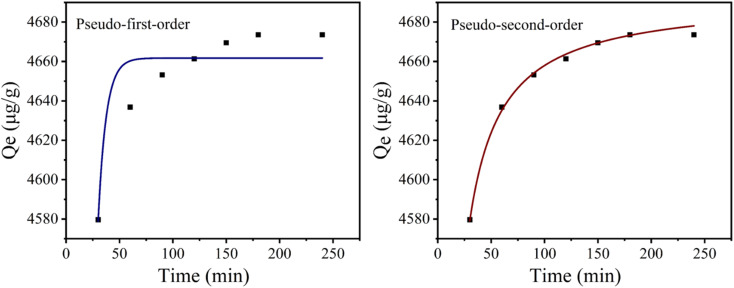
Adsorption kinetics model of phosphate by CoAl-LDH/CeO_2_.

Fig. 7 showed the kinetic curves of phosphate adsorption by CoAl-LDH/CeO_2_. It was worth noting that when the adsorbent was added into the phosphate solution, phosphate was quickly captured. After 2 h, the adsorption processes slowed down and achieved equilibrium, owing to an integrated result of the exhaustion of the less absorbable anions and available adsorption sites in bulk solution. Pseudo-second-order kinetic model for the phosphate adsorption process was better than the pseudo-first-order kinetic model (Table 3). Besides, the absorption capacity determined through fitting nearly accorded with the measured value at equilibrium, and chemical adsorption dominated the entire adsorption process. Therefore, this CoAl-LDH/CeO_2_ was a fast and efficient adsorbent.

**Table 3:** Kinetics parameters of phosphate onto CoAl-LDH/CeO_2_

*C*_0_ (μg mL^−1^)	*q*_e,cal_ (μg g^−1^)	Pseudo-first-order			Pseudo-second-order		
		*q*_e,cal_ (μg g^−1^)	*K*_1_ (min^−1^)	*R* ^2^	*q*_ecal_ (μg g^−1^)	*K*_2_ (g μg^−1^ min)	*R* ^2^
20	4673.61	4661.7	0.1343	0.8286	4692.5	2.8667	0.9956

### 3.4. Conditions and influence of parameters on adsorption process

**Fig. 8:**
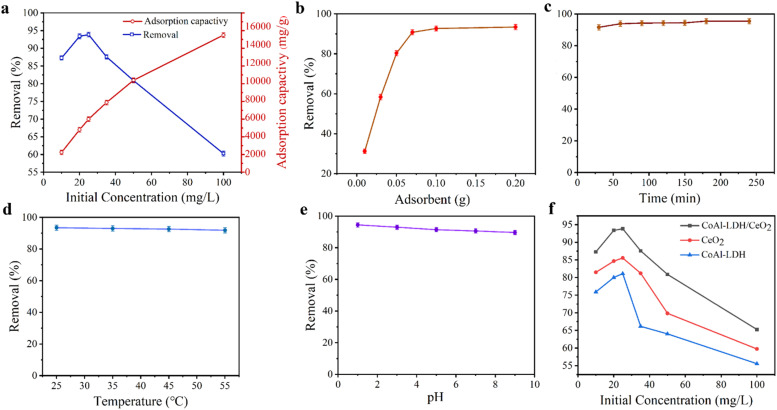
(a) Effect of initial concentration on removal rate of CoAl-LDH/CeO_2_, (b) effect of CoAl-LDH/CeO_2_ dosage on adsorption properties, (c–e) effect of time, temperature and initial solution pH on adsorption properties, respectively, (f) comparison of the adsorption effect of CoAl-LDH, CeO_2_ and CoAl-LDH/CeO_2_ on phosphate.

Firstly, the effect of initial phosphate concentration on the adsorption capacity of CoAl-LDH/CeO_2_ was determined, as displayed in Fig. 8a. The results showed that the phosphate removal rate reached to 93.4% as the initial concentration was 20 mgL^−1^. After that, the removal rate gradually decreased with the concentration increasing. In general, adsorption involved the surface reaction processes, thus the initial adsorption rate was relatively fast. The phosphate absorption extent was remarkably reduced, which was connected to the diminution of surface vacancies. After a period of time, the remaining vacancies were difficult to occupy, because of the repulsive effect between solute molecules on the solid surface and the bulk phase. Therefore, the phosphate concentration of 20 mgL^−1^ was employed as the initial concentration for the following experiment.

Secondly, the effect of adsorbent dosage on the phosphate adsorption performance for CoAl-LDH/CeO_2_ composites was demonstrated in Fig. 8b. At the beginning, the removal rate of phosphate obviously increased with the amount of adsorbent gradually increasing. When the dosage of adsorbent was 0.10 g, the removal rate reached 92.6%. Subsequently, with the amount of adsorbent increasing, the removal rate increased slowly and tended to flatten out. The reason may be that the concentration of phosphate ions reduced, and the residual phosphate ions permeated more slowly, resulting in a decrease in adsorption efficiency.^52^ If economic factors were considered, the optimal dosage of CoAl-LDH/CeO_2_ in phosphate solution treatment was 0.10 g.

Thirdly, the effects of contact time (Fig. 8c) and temperature (Fig. 8d) on adsorption were investigated in the same batch system. Initially, the phosphate adsorption rate on adsorbents got 91.6% within 30 minutes. Then, it slowly and gently increased, reaching 93.8% within 60 minutes. The results revealed that with the increase of time, the phosphate removal rate increased great slowly until 120 minutes, indicating that the system has reached equilibrium. Because of the sufficient adsorption sites on the outer surface of CoAl-LDH/CeO_2_, phosphate adsorption was fast in the early stages and could complete in a short time. It suggested that CoAl-LDH/CeO_2_ had the competence to quickly remove pollutants. Therefore, the optimal adsorption time was 60 minutes. The effect of temperature on adsorption (see the Fig. 8d) confirmed that temperature had no significant effect on adsorption, thus CoAl-LDH/CeO_2_ can adsorb phosphate in the range of 15 °C to 45 °C.

The pH of the solution is a key factor which affects the adsorption efficiency of target pollutants. Fourthly, the effect of initial pH value in phosphate solution adsorbed onto each adsorbent at equilibrium was executed, and the results generally were displayed in Fig. 8e, which showed that the phosphate removal efficiency for CoAl-LDH/CeO_2_ gradually decreased from 94.4% to 89.6%, while the pH value of solution increased from 1.0 to 9.0, but its decline was not very obvious. The reason may be that phosphate interleaved in CoAl-LDH/CeO_2_ needed to be released with a more durable way in an acidic solution. When the pH value increased, the positive and negative charges on the superficies of CoAl-LDH/CeO_2_ diminished. Additionally, at higher pH values, on the adsorbent surface of CoAl-LDH/CeO_2_, more OH– would compete with phosphate for the equivalent adsorption active sites, which made for a decrease for the removal capability of phosphate by the adsorbent.^53^ This represented that low pH values are beneficial for adsorption, and the capacity will be higher at low pH values. Therefore, pH was not regulated in the following experiment. All in all, the wide operating pH range of CoAl-LDH/CeO_2_ favoured for its industrial application.

Finally, the adsorption capability of CoAl-LDH/CeO_2_ was compared with CoAl-LDH and CeO_2_. As displayed in Fig. 8f, after only 60 minutes of phosphate adsorption by CoAl-LDH/CeO_2_, the removal rate reached 93.4%, which was higher than CoAl-LDH (84.6%) and CeO_2_ (80.0%). The CoAl-LDH/CeO_2_ displayed an ultrafast kinetic process and ultrahigh removal effect for phosphate adsorption. In summary, the addition of CoAl-LDH to induce the synthesis of CoAl-LDH/CeO_2_ nanomaterials resulted in a demonstrable improvement in the adsorption efficiency of phosphate. The adsorption results suggested that the phosphate removal capacity dramatically ameliorated with the increase of CoAl-LDH in the nano combination. The more specific surface area and more uniform nanosphere structure of hydrotalcite-decorated nano-CeO_2_ based combination (CoAl-LDH/CeO_2_) was able to improve the adsorption efficiency of phosphate.

### 3.5. Effect of Co-existing ions

**Fig. 9:**
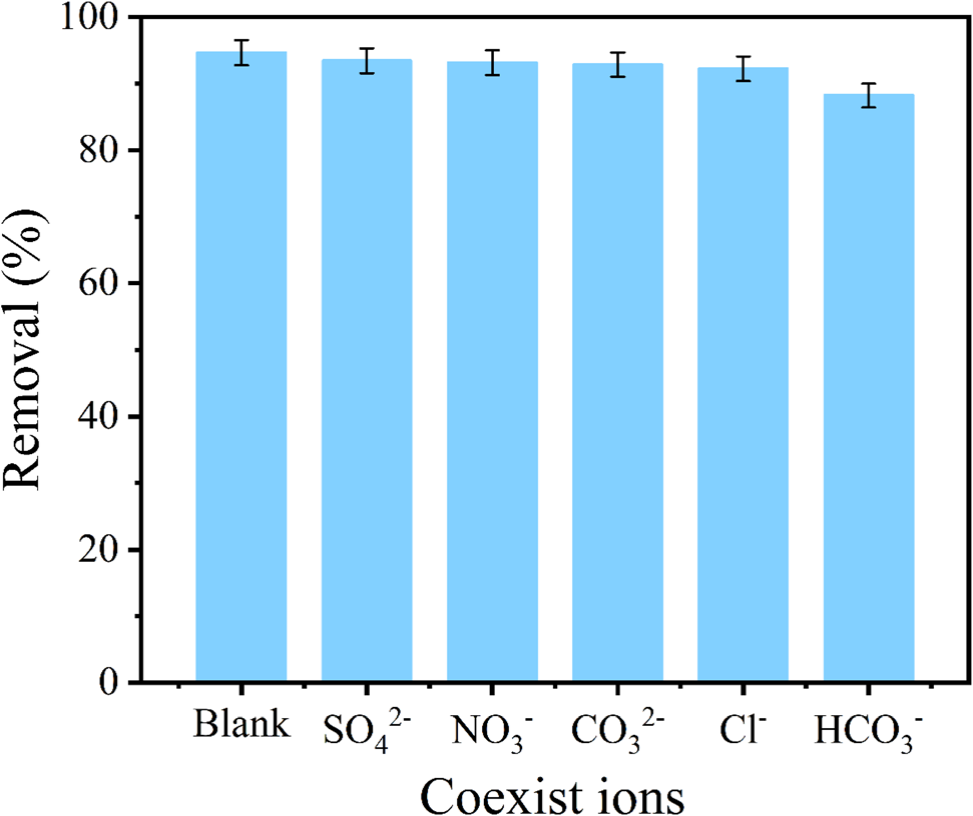
Effect of coexisting anions (SO_4_^2−^, Cl^−^, NO_3_^−^ and CO_3_^2−^, HCO_3_^−^) on the adsorption of phosphate by CoAl-LDH/CeO_2_.

As well known, phosphate normally coexisted together with other anions in actual effluent. Accordingly, the phosphate selectivity of CoAl-LDH/CeO_2_ was great significant. As exhibited in Fig. 9, it could be obviously seen that the influence of the coexisting anions (SO_4_^2−^, Cl^−^, NO_3_^−^, CO_3_^2−^, and HCO_3_^−^) on the phosphate capture by CoAl-LDH/CeO_2_ were examined. The results displayed that a negligible effect of the adsorption capacity and removal efficiency was confirmed even the phosphate concentration was much lower than those of the coexisting anions (Cl^−^, NO_3_^−^, CO_3_^−^ and SO_4_^2−^), exhibiting that CoAl-LDH/CeO_2_ showed specific recognition of phosphate by adsorption sites when CO_3_^2−^, NO_3_^−^, Cl^−^ and SO_4_^2−^ coexisted in the water solution. However, the phosphate removal rate decreased slightly from 93.63% to 88.43% with the presence of 0.01 M HCO_3_^−^. This phenomenon could be illustrated that Cl^−^, NO_3_^−^ and SO_4_^2−^ can only form an outer complex with the adsorbent during the adsorption process, which hardly affected the adsorption of phosphate by the adsorbent. However, HCO_3_^−^ can combine with most adsorbents to form a strong innerlayer complexes, leading to a significant decrease in the adsorption capacity of phosphate on CoAl-LDH/CeO_2_.^54^

### 3.6. Recycling performance

**Fig. 10:**
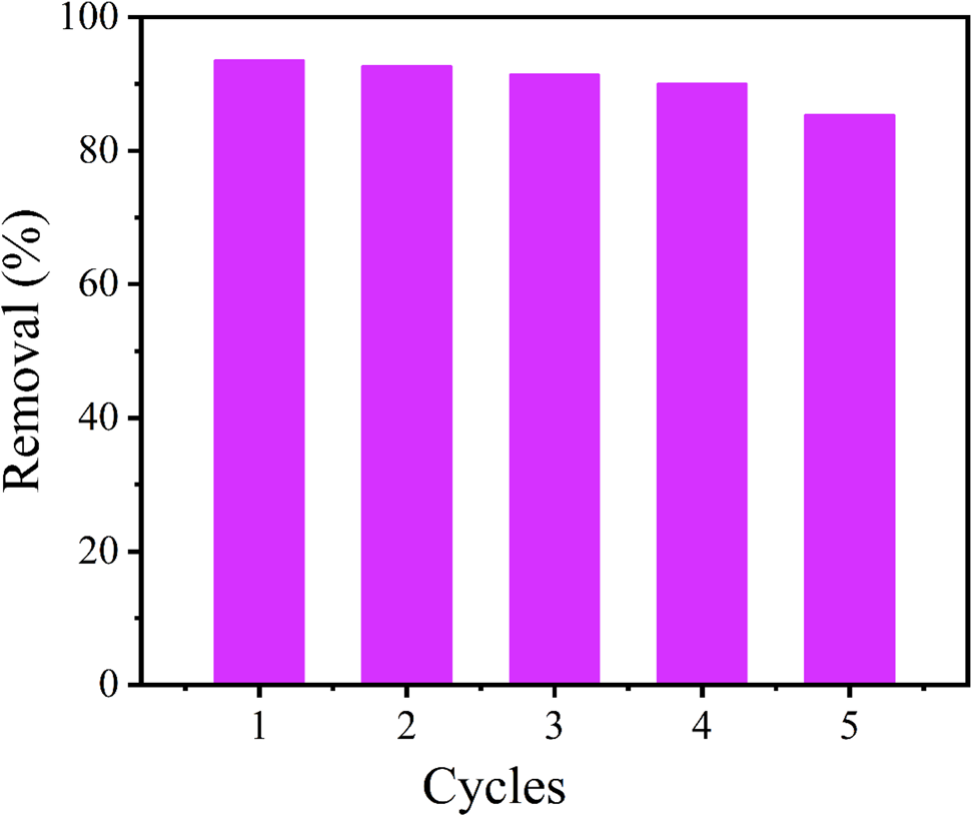
Reuse performance ratio of CoAl-LDH/CeO_2_ adsorbed phosphate.

The cycling behavior of CoAl-LDH/CeO_2_ was crucial for industrial applications. In order to study the reusability of CoAl-LDH/CeO_2_, six recursive adsorption–desorption experiments were conducted. As displayed in Fig. 10, after six cycles, the phosphate removal rate of CoAl-LDH/CeO_2_ was still higher than 85%, indicating that most phosphate adsorbed on CoAl-LDH/CeO_2_ could be desorbed and reclaimed through NaOH solution. In addition, it was distinctly investigated that the phosphate removal rate gradually decreased with the number of cycles increasing, due to the loss of adsorbent during the recycling process bringing about a reduction in adsorption sites. Nevertheless, the phosphate capture ability of CoAl-LDH/CeO_2_ was able to maintain at 85.3% after six cycles, indicating that CoAl-LDH/CeO_2_ had a considerable reclaiming performance.

### 3.7. Comparing the CoAl-LDH/CeO_2_ with different adsorbents

The performance of CoAl-LDH/CeO_2_ in absorbing phosphate was compared to other materials reported in the literature (Table 4). The CoAl-LDH/CeO_2_ composite was found to had a high capacity to absorb phosphate ions from aqueous solutions based on this data. This may be attributed to the presence of ceria nanoparticles on the surface of CoAl-LDH, which can act as active sites to capture phosphate ions from contaminated aqueous solutions. Therefore, CoAl-LDH/CeO_2_ could be considered as the top and most effective adsorbent for purifying water from low concentration phosphate pollutants.

**Table 4:** Comparing the CoAl-LDH/CeO_2_ for phosphate – uptake with different absorbents in the reported literature

Adsorbent	Temp (°C)	Adsorption capacity (mg g^−1^)	Ref.
Hybrid anionic exchanger (HAIX)	23	2.25 mg g^−1^	55
CaT-Z	37	8.79 mg g^−1^	56
Marine macroalgae BC	20	3.3 mg g^−1^	57
SDS-GO	25	3.9 mg g^−1^	58
Magnetite-based nanoparticles	25	5.2 mg g^−1^	59
CoAl-LDH/CeO_2_	25	16.03 mg g^−1^	This work

### 3.8. Possible mechanism of the CoAl-LDH/CeO_2_ surface

**Scheme 1:**
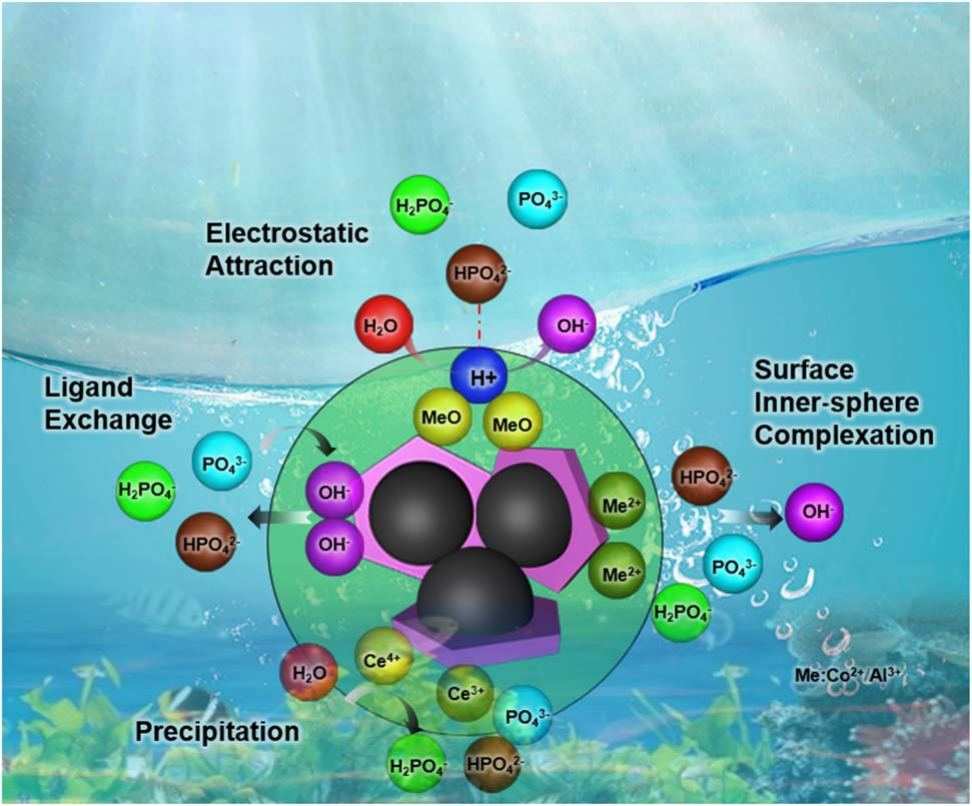
A schematic diagram of the adsorption mechanism by CoAl-LDH/CeO_2_.

The mechanism of phosphate removal on the surface of CoAl-LDH/CeO_2_ nanocomposite was proposed by a redox reaction that takes place between ceria nanoparticles on the surface of the CoAl-LDH/CeO_2_ adsorbent and phosphate ions in the contaminated water solution to generate CePO_4_ on the adsorbent surface. The results displayed both spectra had Ce 3d3/2 and Ce 3d5/2 spin–orbit splitting which demonstrated the presence of Ce^3+^and Ce^4+^ oxidation states on the CoAl-LDH/CeO_2._ There are literature reports on the redox reactions that have occurred since Ce^3+^was synthesized on adsorbents (CoAl-LDH/CeO_2_), where Ce^3+^was oxidized to Ce^4+^and CeO_2_ nuclei began to form after heat treatment. However, in the presence of phosphate, the reduction from Ce^4+^ to Ce^3+^ occurred on the adsorbent (CoAl-LDH/CeO_2_), and CePO_4_ formed by the reaction of Ce^3+^ and phosphate to generate CePO_4_.^60^ Therefore, the distributed CeO_2_ on the surface of CoAl-LDH exerted a major role in phosphate ions uptake from an aqueous solution by the oxidation, reduction, and ion-exchange of adsorbed Ce^3+^ on the CoAl-LDH from CeO_2_ to CePO_4_. After the addition of CoAl-LDH, the complex not only had the adsorption of CeO_2_, but also the adsorption of CoAl-LDH. According to literature result, it was suggested that the adsorption of phosphate by LDH was controlled by electrostatic attraction, ligand exchange, intrasphere complexation and precipitation.^61^ At the beginning of phosphate adsorption with pH range of 1.0–9.0, electrostatic attraction between H_2_PO_4_^2−^ and positively charged CoAl-LDH was expected. CoAl-LDH/CeO_2_ before adsorption was pH-dependent positive charge surface, while the zeta potentials of CoAl-LDH were gradually decreased until negative charge surface (Fig. 3d). The overall adsorption mechanism of CoAl-LDH/CeO_2_ for phosphate removal can be described by the Scheme 1.

## 4. Conclusions

In conclusion, this study explored the novel adsorbent CoAl-LDH/CeO_2_ according to the synthetic tactics. The decorating morphology and particle size of CoAl-LDH/CeO_2_ were obtained with CeO_2_ modified by CoAl-LDH nanosheets. It is worth noting that the addition of CoAl-LDH not only promoted the uniform distribution of CeO_2_, but also reduced its own agglomeration. Amazingly, the removal rate of phosphate by CoAl-LDH/CeO_2_ could reach 91.67% at room temperature within 30 minutes, demonstrating its ultrafast and high adsorption efficiency, while the final concentration of the phosphate solution decreased to 1.68 μg P L^−1^, far below the US Environmental Protection Agency's emission standards (50 μg P L^−1^).^62^ Furthermore, when Cl^−^, NO_3_^−^, SO_4_^2−^*etc.* coexisted in the solution, CoAl-LDH/CeO_2_ still possessed favourable selectivity for phosphates. Phosphate capture capacity of CoAl-LDH/CeO_2_ could be maintained over 85% after six cycles. The greatly enhanced adsorption activity could be attributed to the morphology of CoAl-LDH/CeO_2_ composites contented with hexagonal platelets of CoAl-LDH and CeO_2_ nanospheres. In a word, the CoAl-LDH/CeO_2_ composite displayed an outstanding adsorption property toward low concentration phosphate, thus indicating that it is a potential material in the field of secondary wastewater treatment. It had the potential application in remediation of water containing phosphate.

## Author contributions

Fengqin Tang: visualization, methodology, data curation, formal analysis, writing – original draft preparation. Hui Bai: investigation, conceptualization, methodology, data curation, formal analysis. Yahui Chen: article polishing, formal analysis. Wenyuan Liu: methodology, software. Chunhui Shi: editing. Lisheng Zhang: software. Yaju Zhang: conceptualization. Ling Yang: writing – review & editing. Libing Hu: funding acquisition, project administration, supervision, writing – review & editing.

## Conflicts of interest

There are no conflicts to declare.

## Supplementary Material

## References

[cit1] Yang W., Zhang Y., Zheng J., Liu L., Si M., Liao Q., Zhao F. (2023). Chemosphere.

[cit2] Hong S. P., Yoon H., Lee J., Kim C., Kim S., Lee J., Yoon J. (2020). J. Colloid Interface Sci..

[cit3] Karl D. M. (2000). Nature.

[cit4] Li H., Cui S., Tan Y., Peng Y., Gao X., Yang X., Chen Q. (2022). Environ. Pollut..

[cit5] Wu Y., Li X., Yang Q., Wang D., Xu Q., Yao F., Huang X. (2019). J. Environ. Manage..

[cit6] Shang Y., Guo K., Jiang P., Xu X., Gao B. (2018). Int. J. Biol. Macromol..

[cit7] Liu L., Yang Z., Zhao F. (2023). Chem. Eng. J..

[cit8] Lin J., He S., Zhan Y., Zhang H. (2020). Environ. Technol..

[cit9] Xu C., Feng Y., Li H., Yang Y., Wu R. (2023). Chemosphere.

[cit10] Kim D., Kim J., Lee K. W., Lee T. S. (2019). Microporous Mesoporous Mater..

[cit11] Leyva-Ramos R., Ocampo-Pérez R., Bautista-Toledo I., Rivera-Utrilla J., Medellín-Castillo N. A., Aguilar-Madera C. A. (2020). Chem. Eng. Commun..

[cit12] Luo D., Wang L., Nan H., Cao Y., Wang H., Kumar V. T., Wang C. (2023). Environ. Chem. Lett..

[cit13] Zhao Z., Wang B., Feng Q., Chen M., Zhang X., Zhao R. (2023). Sci. Total Environ..

[cit14] He J., Xu Y., Wang W., Hu B., Wang Z., Yang X., Yang L. (2020). Chem. Eng. J..

[cit15] Huang C., Zhang H., Zheng K., Zhang Z., Jiang Q., Li J. (2021). Sci. Total Environ..

[cit16] Yang L., Shan X., Zhao Y., Xiao Z., An Q., Zhai S. (2022). Microporous Mesoporous Mater..

[cit17] Shan X., Zhao Y., Bo S., Yang L., Xiao Z., An Q., Zhai S. (2021). Sci. Total Environ..

[cit18] Liu X., He X., Zhang J., Yang J., Xiang X., Ma Z., Zong E. (2020). RSC Adv..

[cit19] Ko Y. G., Do T., Chun Y., Kim C. H., Choi U. S., Kim J. Y. (2016). J. Hazard. Mater..

[cit20] Lu H., Feng Y., Feng Y., Dong Y., Sun H., Xing J. (2019). Geoderma.

[cit21] Bakry A. M., Alamier W. M., Salama R., Samy El-Shall M., Awad F. (2022). Surf. Interfaces.

[cit22] Liu L., Yang Z., Yang W., Jiang W., Liao Q., Si M., Zhao F. (2024). J. Environ. Sci..

[cit23] Khan A. I., O'Hare D. (2002). J. Mater. Chem..

[cit24] Sohrabi H., Khataee A., Ghasemzadeh S., Majidi M. R., Orooji Y. (2021). Trends Environ. Anal..

[cit25] Ghasemi M., Khataee A., Gholami P., Soltani R. D. C., Hassani A., Orooji Y. (2020). J. Environ. Manage..

[cit26] Aref-Oskoui S., Khataee A., Vatanpour V. (2017). ACS Comb. Sci..

[cit27] Li L., Gu W., Chen J., Chen W., Xu Z. P. (2014). Biomaterials.

[cit28] Goh K. H., Lim T. T., Dong Z. (2008). Water Res..

[cit29] Sheng T., Zhang Z., Hu Y., Tao Y., Zhang J., Shen Z., Feng J., Zhang A. (2019). Environ. Sci. Pollut. Res..

[cit30] Buates J., Imai T. (2020). J. Water Process Eng..

[cit31] Tang F., Yang H., Chen H., Zhou M., Huang P., He Y., Song P., Wang R. (2022). J. Environ. Chem. Eng..

[cit32] Jia Y., Zhang Y., Fu J., Yuan L., Li Z., Liu C., Wang X. (2019). Colloids Surf., A.

[cit33] Wan S., Wang S., Li Y., Gao B. (2017). J. Ind. Eng. Chem..

[cit34] Channei D., Chansaenpak K., Phanichphant S., Jannoey P., Khanitchaidecha W., Nakaruk A. (2021). ACS Omega.

[cit35] Deus R. C., Cilense M., Foschini C. R., Ramirez M. A., Longo E., Simões A. Z. (2013). J. Alloys Compd..

[cit36] Hsieh S. H., Manivel A., Lee G. J., Wu J. J. (2013). Mater. Res. Bull..

[cit37] Rong M., Yang F., Yu C., Wang S., Zhong H., Cao Z. (2020). Colloids Surf., A.

[cit38] Zhu Y., Dong X., Cheng J., Wang L., Zhao C., Deng Y., Xie S., Pan Y., Zhao Y. (2023). Chin. Chem. Lett..

[cit39] Chen Y., Jing C., Zhang X., Jian D., Liu X., Dong B., Fen L., Li S., Zhang Y. (2019). J. Colloid Interface Sci..

[cit40] Ali S., Jian Y., Lai Z., Zhang P., Ye S., Wang J., Fu J., Zhang N., Zheng J., Chen B. (2023). J. Rare Earths.

[cit41] Kang D., Yu X., Ge M. (2017). Chem. Eng. J..

[cit42] Zhang Y., Du D., Li X., Sun H., Li L., Bai P., Xin W., Xue Q., Yan Z. (2017). ACS Appl. Mater. Interfaces.

[cit43] Fu J., Xu Q., Low J., Jiang C., Yu J. (2019). Appl. Catal., B.

[cit44] Panchal D., Sharma A., Mondal P., Prakash O., Pal S. (2021). Appl. Surf. Sci..

[cit45] Jiang Y., Guo J., Li X., Wu G., Mu M., Yin X. (2022). Sol. Energy.

[cit46] Younis A., Shirsath S. E., Shabbir B., Sean L. (2018). Nanoscale.

[cit47] Guo X., Fan Z., Wang Y., Jin Z. (2021). Surf. Interfaces.

[cit48] Zhang W., Wang Z., Zhao Y., Miras H. N., Song Y. (2019). ChemCatChem.

[cit49] Ramutshatsha-Makhwedzha D., Mavhungu A., Moropeng M. L., Mbaya R. (2022). Heliyon.

[cit50] Xie Y., Fan L., Liu W., Zhang Q., Huang G. (2023). Particuology.

[cit51] He F., Yang Z., Zhao F., Repo E., Yang W., Liao Q., Lin Z. (2023). Environ. Sci.: Nano.

[cit52] Simoes dos Reis G., Cazacliu B. G., Correa C. R., Ovsyannikova E., Kruse A., Sampaio C. H., Dotto G. L. (2020). J. Environ. Chem. Eng..

[cit53] Trazzi P. A., Leahy J. J., Hayes M. H. B., Kwapinski W. (2016). J. Environ. Chem. Eng..

[cit54] Wang Y., Xie X., Chen X., Huang C., Yang S. (2020). J. Hazard. Mater..

[cit55] Blaney M. L., Cinar S., SenGupta K. A. (2007). Water Res..

[cit56] Mitrogiannis D., Psychoyou M., Baziotis I., Inglezakis J. V., Koukouzas N. (2017). Chem. Eng. J..

[cit57] Jung W. K., Ahn H. K. (2016). Bioresour. Technol..

[cit58] Bakry M. A., Alamier M. W., Salama S., El-Shall S. M., Awad S. F. (2022). Surf. Interfaces.

[cit59] Daou J. T., Begin-Colin S., Greneche M. J., Thomas F., Derory A., Bernhardt P. (2007). Chem. Mater..

[cit60] Ko G. Y., Do T., Chun Y., Kim H. C., Choi S. U., Kim Y. J. (2016). J. Hazard. Mater..

[cit61] Zhang X., Shen J., Ma Y., Liu L., Meng R., Yao J. (2020). J. Colloid Interface Sci..

[cit62] Jiao G., Ma J., Zhang Y., Jin D., Li Y., Hu C., Sun R. (2021). Int. J. Biol. Macromol..

